# Preferences for public involvement in health service decisions: a comparison between best-worst scaling and trio-wise stated preference elicitation techniques

**DOI:** 10.1007/s10198-016-0856-4

**Published:** 2016-12-10

**Authors:** Seda Erdem, Danny Campbell

**Affiliations:** Economics Division, Stirling Management School, University of Stirling, Stirling, UK

**Keywords:** Trio-wise, Best-worst scaling, Stated preference elicitation, Public health, Public involvement

## Abstract

Stated preference elicitation techniques, such as discrete choice experiments and best-worst scaling, are now widely used in health research to explore the public’s choices and preferences. In this paper, we propose an alternative stated preference elicitation technique, which we refer to as ‘trio-wise’. We explain this new technique, its relative advantages, modeling framework, and how it compares to the best-worst scaling method. To better illustrate the differences and similarities, we utilize best-worst scaling Case 2, where individuals make best and worst (most and least) choices for the attribute levels that describe a single profile. We demonstrate this new preference elicitation technique using an empirical case study that explores preferences among the general public for ways to involve them in decisions concerning the health care system. Our findings show that the best-worst scaling and trio-wise preference elicitation techniques both retrieve similar preferences. However, the capability of our trio-wise method to provide additional information on the strength of rank preferences and its ability to accommodate indifferent preferences lead us to prefer it over the standard best-worst scaling technique.

## Introduction

Understanding public priorities and preferences relating to policy options and investment opportunities is central to policy appraisal. Problematically though, eliciting public opinion and preferences relating to the design and application of public involvement activities in health care services is not straightforward. The policy options are numerous, and hence a multi-criteria approach is warranted. Various approaches can be used to rank the characteristics of public involvement activities in terms of their importance to the public. A straightforward approach is to ask respondents to compare items in a list and to identify them in order of preference. However, while people can usually comfortably rank a small list of items, as the list of items increases, the ranking task becomes increasingly overwhelming, ultimately, requiring additional cognitive effort over a longer duration.

As an alternative to asking respondents to provide a complete ranking of the items, Finn and Louviere [1] proposed the best-worst scaling (BWS) technique, whereby respondents choose two items from a subset of the list in terms of their underlying scale of importance (e.g., best and worst, or most and least important). This technique is an extension of Thurstone’s [2] method of pair-wise comparison, which has the idea of eliciting trade-offs between paired items. BWS is a more general version of the method of paired comparison since it allows the comparison of more than two items in a task in which a respondent chooses the ‘best’ and ‘worst’ (or ‘most’ and ‘least’) items. The appealing feature of the BWS technique is that respondents only need to identify their extreme ranks, rather than indicating the level of their preferences on a scale, such as “somewhat preferred” and “extremely preferred”. This has the potential to reduce—if not eliminate—many of the anomalous behaviors associated with ranking a large number of items (e.g., fatigue, scale-use bias). There is also some evidence that the predictive power of BWS in eliciting preferences is superior compared those produced from rating tasks via the use of Likert scales [3, 4]. Not surprisingly, this technique has become a widely accepted approach for exploring stated preferences in health research (e.g., see [5–8] for recent examples). Notwithstanding these advantages, the BWS method can be restrictive in cases where respondents consider two or more of the items to be either most or least important at a choice task. Although ties in preferences may be established by comparing choices across a sequence of BWS choice tasks, in a typical single BWS choice task, respondents are confined to expressing the items that they most and least prefer.

In this paper, we propose a new stated preference elicitation technique, which we call ‘trio-wise’, as an alternative to the BWS methodology. Unlike BWS, where respondents are presented a horizontal/vertical list of items, we present the choice task in the form of an equilateral triangle. With each vertex of the triangle representing a specific item, respondents are required to identify the location on the triangle that best describes their ranking for the three presented items. The closer a respondent clicks to one of the vertices, the more they prefer the item associated with that vertex as compared to the items represented by the two remaining vertices. Respondents are permitted to click on any point on the triangle. However, compared to a BWS choice task comprising of three items, the trio-wise preference elicitation method produces additional insight relating to the strength (i.e., intensity) of a respondent’s rankings and preferences. This means that the same level of information can be recouped from respondents using fewer choice tasks. A further advantage of the trio-wise method is that it can accommodate instances where respondents have indifferent preferences for items presented in a given choice task. Arguably, this may lead to more reliable preferences, since respondents are not coerced into choosing a best and a worst item, perhaps at random, when their preferences for many of the items are indistinguishable. Moreover, allowing respondents to express their indifference may even help reduce frustration associated with indicating their extreme rankings.

The aim of this paper is to introduce the new trio-wise technique and to compare it with the widely used BWS approach. To do so, we use an empirical case study that explores preferences among the UK general public for ways to involve them in decisions affecting the national health care system. We explore the extent to which rankings and policy repercussions are consistent across the two preference elicitation methods under different model specifications.

## Methods

We begin this section by outlining the BWS approach and by describing our trio-wise stated preference elicitation approach. We then introduce our empirical case study and modeling approach.

### Best-worst scaling stated preference elicitation method

The BWS approach is one type of stated preference techniques that are now routinely used to prioritize resources in health care systems. This technique was developed by Finn and Louviere [1] as an extension of Thurstone’s [2] method of pair-wise comparison. As part of this method, respondents are shown a subset of a list of items and are asked to identify the two items in the subset that maximize the difference between them on an underlying scale (e.g., best and worst, or most and least important). Respondents face a sequence of such choice tasks, each of which includes a different subset from the list. The full ranking of all items under investigation is then retrieved by analyzing the panel of choices.

The BWS technique is particularly suited when preferences are sought for a large number of items. This stems from the fact that the ranking task is broken down into a sequence of smaller, and more manageable, tasks. This significantly reduces cognitive effort, since it avoids respondents having to rank the full list of items at one instance. In such cases, measurement error is likely to be relatively high, and the choices may be more prone to anomalous behavior [9]. Furthermore, BWS does not suffer from the scale-use bias that has been found when preferences are measured using Likert-based scales [3, 4, 10]. An appealing feature of the BWS technique is that respondents only have to identify their extreme preferences (e.g., best/worst, most/least) and this tends to be easier and lead to better judgments [3]. These appealing features of the BWS technique have increased its application not only in health research (e.g., [7, 8, 11]), but also in a range of disciples, including environment and agriculture (e.g., [9, 12, 13]).

Since respondents only reveal their extreme preferences, the BWS approach does not require respondents to provide any information about the intensity of their preferences. In many situations, however, it is desirable to give respondents the opportunity to express some measure of intensity of preference. Indeed, overlooking this could led to erroneous policy recommendation, since respondents’ preferences are not appropriately reflected. To illustrate this, suppose that slightly over half of respondents consider item 1 to be very marginally more important than item 2, but the remaining respondents deem item 2 as being hugely more important relative to item 1. Based on the BWS framework, the predicted ordinal ranking of the items would be item 1 followed by item 2, while it is clear that when the intensity of preferences are accounted for the consensus ranking will be reversed.

As described in Louviere et al. [14], there are three types (cases) of BWS, which largely differ in the complexity of the choice items offered: (1) object case; (2) profile case; and, (3) multi-profile case. Case 1 presents items that have no attributes or levels (e.g., attitudinal statements). Case 2, which is widely used within health research, requires respondents to make best and worst (most and least) choices for the attribute levels that describe a single profile. For example, the choices would be the most important and least important features (i.e., attribute levels) of a public health policy (i.e., profile). Case 3 consists of at least three profiles that are described using different attribute levels. In this sense, BWS Case 3 is simply an extension of the more familiar discrete choice experiment methodology, but instead of asking respondents to select only the profile that they most prefer, respondents are also asked to identify the profile that they least prefer, which make the decision more complex compared to the Cases 1 and 2. As a proof of concept, we use the BWS Case 2 to better illustrate the differences and similarities with the new trio-wise method. However, it is possible to extend this comparison to other BWS cases. Indeed, we note that extending the trio-wise to Case 3 offers an interesting extension.

### Trio-wise stated preference elicitation method

**Fig. 1:**
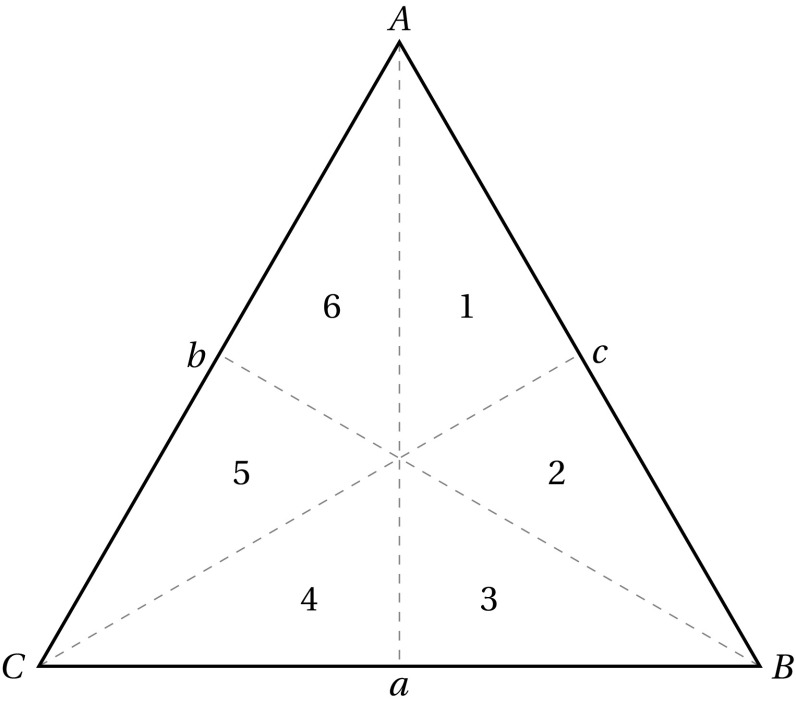
A trio-wise choice task

The trio-wise technique we introduce in this paper as an alternative to BWS has many of the same characteristics of BWS. Crucially, however, it allows respondents to express their measure of preference intensity without using a ranked-based scale (e.g., Likert scale) or the need for any follow-up questions. The method supposes that the choice task can be represented as an equilateral triangle and that rankings are consistent with the relative distances to each vertex associated with a specific item. An example of such a choice task is presented in Fig. 1. Specifically, respondents are instructed to identify the point on the triangle that best describes their ranking for the three different items presented at the vertices. Respondents are permitted to click any point on the triangle. They are informed that the closer they click to a vertex, the more important the item on that vertex becomes for them. As part of the trio-wise approach, the coordinates of the selected point are recorded, and the Euclidean distances to the vertices are measured so that a complete ranking of the three items presented in the trio-wise choice task can be determined. For instance, all points identified left of the angle bisector *Aa* in Fig. 1 imply that Item *C* is preferred over Item B (i.e., $C>B$). Points to the right of this line imply the reverse. Similar inferences can be reached on the basis of whether the point is left/right/above/below the angle bisectors *Bb* and *Cc*. Indeed, the specific ranking order of items can be deduced by knowing in which of the six areas that are formed by partitioning the triangle via the medians the chosen point is located: (1) any point selected within area 1 indicates that $A>B>C$; (2) points within area 2 imply that $B>A>C$; (3) points within area 3 imply that $B>C>A$; (4) points within area 4 imply that $C>B>A$; (5) points within area 5 imply that $C>A>B$; and, (6) points within area 6 imply that $A>C>B$.

**Fig. 2:**
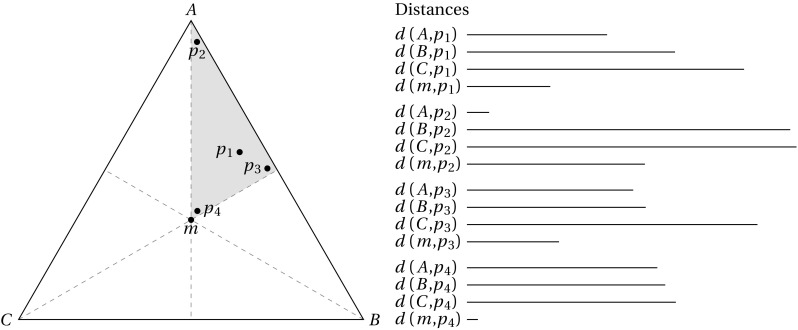
Assessing preference intensity from a trio-wise choice task

The key feature of the trio-wise preference elicitation method is the additional insight it offers relating to the intensity of respondents’ preferences. For example, consider the four chosen points ($p_{1}$, $p_{2}$, $p_{3}$ and $p_{4}$) in Fig. 2. Note that all four points are located within an area associated with the same ordinal ranking (i.e., $A>B>C$). Therefore, in a standard BWS setting, in all four cases, Item *A* and Item *C* would be identified as the most and least preferred item respectively—but no clue would be given as to the degree to which Item *A* and Item *C* are more and less preferred respectively over Item *B* in a choice task. However, in the trio-wise choice task, we can see that the distances between the vertices and the chosen points differ in the four choices. A respondent who clicks on $p_{1}$ has quite distinct preferences for Items *A*, *B* and *C*, as evident in the different lengths of $d( A,p_{1})$, $d( B,p_{1})$ and $d( C,p_{1})$, which are represented by the horizontal lines in Fig. 2. Compare this to a respondent who clicks on $p_{2}$. Noticeably, this respondent holds a much stronger preference for Item *A* relative to Items *B* and *C*, which are both of a similar preference intensity [i.e., $p_{2}$ is relatively equidistant from *B* and *C*, as illustrated by lines $d( B,p_{2})$ and $d( C,p_{2})$]. A respondent who clicks on $p_{3}$ undoubtedly considers Item *C* as being inferior. The distances to vertices *A* and *B*, however, are quite similar, indicating relative indifference between these two items. Although a respondent who clicks on $p_{4}$ also holds the same ordinal ranking as other points, we can infer that their strength of preferences for items A, B and C is much weaker [i.e., $d( A,p_{4})$, $d( B,p_{4})$ and $d( C,p_{4})$ are all of relatively similar length]. This means that the preferences for the three items are relatively aligned. In other words, Item *A* is only slightly preferred over Item *B*, which is only marginally preferred over Item *C*. This ability to simultaneously establish preference intensity is important for another reason. It means that the same level of information can be recouped from respondents using fewer choice tasks. With fewer questions, cognitive effort and survey length can be potentially reduced, which, in turn, can decrease the incidence and effects of respondent fatigue [15].

An additional distance measure can be extracted from the coordinates of the selected point. This relates to how far it is from the centroid, *m*. From Fig. 2, it is apparent that this length is different for the four choices. It is also clear that this distance gives a direct measure of the strength of preferences: the line $d( m,p_{2})$ is considerably longer (where preference intensity is relatively strong) compared to the line $d ( m,p_{4} )$ (where preference intensity is relatively weak).

Another important attraction of the trio-wise method over standard BWS is that it allows for indifferent preferences at the choice task level. Although ties in preferences may be established by comparing choices across a sequence of BWS choice tasks, in a typical single BWS choice task respondents are confined to expressing the items that they most and least prefer. This is restrictive in cases where respondents consider two or more of the items to be either most or least important. This is not the case in the trio-wise: instances where respondents are indifferent between two or more of the items listed in the choice task can be accommodated. Respondents are free to click anywhere on the triangle respondents. Referring back to Fig. 1, a respondent who clicks anywhere along the angle bisector *Aa* reveals that they have equal preferences for Items *B* and *C* (above the centroid implies that $A>B=C$, whereas below the centroid $B=C>A$ can be deduced). Likewise, points clicked along angle bisectors *Bb* and *Cc* indicate indifferent preferences between Items *A* and *C* in the former and Items *A* and *B* in the latter. Further more, a three-way tie (i.e., $A=B=C$) in preference ranking can be inferred when the centroid point is clicked. Allowing for indifference is advantageous. Arguably, it should lead to more reliable preferences, since respondents are not coerced into choosing a best and worst item, perhaps at random, when their preferences for many of the items are indistinguishable. This is clearly important when the objective of the preference elicitation study is to inform policy. Moreover, by giving respondents greater flexibility in choice and the opportunity to reveal their underlying rankings, this may even help reduce respondent frustration.

As an aside, we also draw attention to some of the features that the trio-wise approach shares with other methods, such as visual analogue scale and pair-wise comparison methods. They all require the positioning of a point on a scale with known anchors, meaning that the distance between the items can be interpreted as a measure of preference score. Although there are a number of proponents of some of these techniques, who argue that cardinal measures can be used for “strength of preferences” [16, 17], there are many opposing arguments relating to their theoretical validity, which we believe differentiates them from our trio-wise method. Some of these methods do not present a choice and, therefore, lack any opportunity costs, which, arguably, means that they are less well suited for measuring preference strengths [18]. However, our trio-wise method is more analogous to BWS in theory. Specifically, respondents are asked to make a choice between the items presented and this requires them to trade-off between the items. Thus, it also involves opportunity costs.

Notwithstanding the strengths mentioned above, the trio-wise method also has potential weaknesses (many of which also apply to some of the other methods mentioned above). These include potential cognitive burden on respondents when making choices, which may then lead to decision simplification rules (or heuristics), such as position bias. Unlike BWS, the trio-wise method is restricted to presenting three items/profiles per choice task (unless, of course, the choice task is presented as a three-dimensional regular tetrahedron, which, admittedly, would make the choice task even more complicated).

### Case study and study design

Public involvement has been central to decision-making about health services in many countries (e.g., [19, 20]). Evidence has also showed that the public would like to influence health care investment decisions and services [21]. It is also established that public involvement can increase the relevance and appropriateness of health and social care research and contribute towards the quality of the research by accommodating users’ views and opinions [22, 23]. The extent and nature of public involvement in health care varies considerably. It includes participating in clinical decision-making or priority-setting in health care systems, as well as participating in activities such as identifying research topics and questions, giving feedback on research materials, helping in the running of studies and disseminating findings. The means by which such involvement activities take place can also vary markedly: from interviews, focus groups, forums, and structured meetings [24, 25] through to postal and on-line questionnaire methods [26, 27]. Whilst strategies to increase public involvement are well established, it is not always clear how the public would most like to be involved in such decisions. Alongside this, there is a high degree of uncertainty regarding the characteristics of involvement activities that are considered most important to the public. However, to ensure that public involvement and engagement efforts and funding commitments are appropriate, it is necessary to understand how the public rank the features of involvement activities. This is particularly important when there is an aim to engage different groups in the population in health service decision-making.

In this research, we investigate the public’s preferences for ways to involve them in decisions that shape health care priority-setting. Specifically, we investigate the perceived importance of various features of public involvement activities, which vary from brief, face-to-face meetings with a GP, to longer meetings with university researchers about various health care issues, such as how the UK’s Nation Health Service spends its money, or how researchers can design studies to better involve the public. In doing so, we can better establish the types of activities that the public would most likely engage in. In this study, we describe the public involvement activities using the following characteristics:

- the format of the activity (e.g., postal, face-to-face, online);
- who leads the activity (e.g., a doctor, nurse);
- where the activity happens (e.g., a local hospital, university);
- how often the activity happens (e.g., 1–2 times a year, more than 6 times a year);
- how much time the activity requires (e.g., less than 30 min, more than 1 h);
- the impact of the activity (e.g., contributing to health care research, improving existing services);
- the focus of the activity (e.g., local or national issues); and,
- the cost of the activity.

These characteristics were identified from reviews of policy documents and guidances, such as the UK’s National Institute for Health Care Excellence guidelines on public involvement in health care [28, 29], reviews of systematic reviews (e.g., [30–33]), and interviews with health professionals and the general public. Alongside these resources, we also searched the "INVOLVE Database" for applications of public and patient engagements in health care. To ensure clarity and the appropriateness of the public involvement activities and understanding of the BWS and trio-wise choice task, we piloted the questionnaire before fielding. These characteristics were fully described to respondents before they faced the stated preference questions.

This paper compares the views and preferences ascertained from members of the public gathered via two web-based stated preference elicitation surveys. Respondents were allocated to each treatment (study arm) randomly. In the first treatment, respondents answered BWS choice tasks. In the second treatment, respondents completed the trio-wise questions. All other aspects of the web-based surveys were identical. Before presenting the BWS or trio-wise choice tasks to respondents, we provided brief information about the background, concept and items as well as how to answer the questions.

**Fig. 3:**
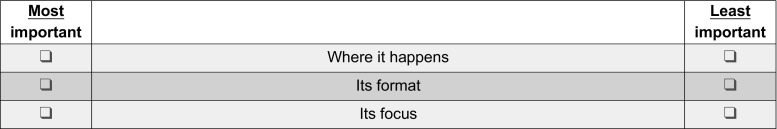
An example of the empirical best-worst scaling task

An example of a BWS task is presented in Fig. 3. So that direct comparisons could be made, each BWS task included three items. Respondents were asked to identify the characteristics of involvement activities in health care that they regarded as being: (1) most important; and, (2) least important.

**Fig. 4:**
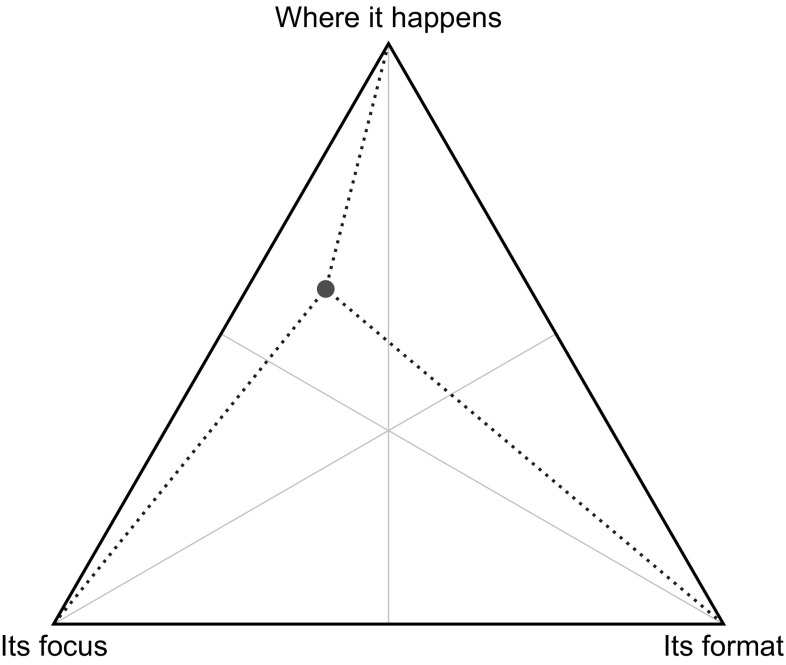
An example of the empirical trio-wise task

In the trio-wise treatment, respondents were presented an equilateral triangle, and where each vertex denoted one of the characteristics of the public involvement activities. An example is given in Fig. 4. Respondents were informed that they could click any point on the triangle,^1^ and that the closer they clicked to a vertex, the more important they considered that characteristic of public involvement to be. Before completing the task they were shown a number of examples to illustrate different rankings and preference intensities. As respondents hovered over the triangle, line segments that joined the vertices and the pointer appeared (as demonstrated in Fig. 4). This allowed respondents to gauge the relative distances and, therefore, accurately express their preference intensities. Respondents could re-click on the triangle as often as they wished, so that they were satisfied with their choice. This interactive choice task was coded using JavaScript programming language. This was tested using different web browsers and screen sizes. While no differences were found across browsers, we, obviously, acknowledge that, despite the choice task occupying most of the screen, the ability to precisely select the preferred point depends on screen size.

#### Experimental design

The BWS and trio-wise choice tasks were generated using the same experimental design, so as to enable meaningful comparison.^2^ The experimental design comprised of five blocks (versions) of nine choice tasks and was generated using the computer-assisted software Ngene [34]. The rationale for multiple blocks was to reduce any context and order effects, which may have reduced the precision of estimates. Each block was generated using a main-effects orthogonal experimental design. The design ensured that the full set of eight characteristics appeared an equal number of times within each block and that the combinations of the three characteristics in these sets satisfied a number of optimal design characteristics, including frequency balance orthogonality, positional balance, and connectivity among tasks. An exploration of the one-way frequencies revealed that the survey design was perfectly balanced as each item in the survey was displayed 17 times across all blocks of the surveys. Moreover, the two-way frequencies showed that the survey had a nearly orthogonal main-effects design, in which each item appeared 4.82 times on average with every other item, with a standard deviation of 0.38. The positional frequencies showed that each item appeared 5.62 times on average in each position, with a standard deviation of 0.48. After ensuring a balanced orthogonal design, the number of choice tasks, survey question framing, and task descriptions were tested using a pilot survey. The order in which the choice tasks were presented to respondents were randomized.

#### Study sample

The web-based surveys were administered in 2014 to a sample of respondents representing the adult (18 years and over) population in the UK. In total, 15,129 BWS observations were collected from 1681 respondents and 15,381 trio-wise observations were collected from 1709 respondents.

Comparison of the BWS and trio-wise samples show no discernible differences in respondents’ characteristics. In both cases, there is an equal split of male and female respondents. The samples also show similarities with regards to the average age (ca. 42 years), income (ca. £22,000 per annum), ethnicity (ca. 92% white) and employment status (ca. 70% employed, 15% unemployed, 12% retired and 7% student). A comparison against the 2011 UK census data suggests that both samples are broadly in line with the UK population.

During the surveys, we also collected information relating to respondents’ experience in public involvement activities, whether they worked in a health care related job or have been a carer. Only 2% in both samples indicated that they were involved in engagement activities before. Less than 10% of both samples said that they currently or previously worked in health care, and a similar proportion in both samples indicated that they are or have been a carer for others.

### Modeling approach

Choices collected from both the BWS and trio-wise stated preference elicitation techniques can be analyzed using the random utility maximization theory framework [2, 35]. In both BWS and trio-wise cases, it is supposed that respondents evaluate all items within the displayed choice task. In the BWS case, a respondent is believed to choose the pair that reflects their maximum difference in ranking, whereas in trio-wise setting, it is assumed that respondents choose a location that reflects their preference intensity for the three items.

#### Best-worst scaling modeling approach

Beginning with the traditional BWS setting, the number of unique pairings of items, which we denote using *J* in a given choice task, is given by $S\left( S-1\right)$, where *S* represents the number of items in a task (i.e., in this study $S=3$). Overall utility, *U*, associated with respondent *n*’s pair choice, *i*, in this task, *t*, is given by the difference in utility between the best and worst items:

1 $$\begin{aligned} U_{nit}= \underbrace{\left( \beta x_{b_{nit}} + \gamma _{b_{nit}}\right) }_{\text {Best}}-\underbrace{\left( \beta x_{w_{nit}} + \gamma _{w_{nit}}\right) }_{\text {Worst}}+\,\varepsilon _{nit}, \end{aligned}$$

where: $\beta$ is a vector of estimated parameters (subject to $\sum \nolimits _{k=1}^{K}\beta _{k}=0$) relating to the best and worst items, *x* (indexed by *b* and *w*, respectively); $\gamma$s are position-specific constants (also indexed by *b* and *w* for best and worst choices) that capture the average effect on utility of all factors that are not included in the model (which are analogous to the alternative-specific-constants that are routinely used in discrete choice modeling and are subject to the constraint $\sum \nolimits _{s=1}^{S}\gamma _{b_{s}}=0$ and $\sum \nolimits _{s=1}^{S}\gamma _{w_{s}}=0$ respectively); and, $\varepsilon$ is an *iid* type I extreme value (EV1) distributed error term, with constant variance equal to $\pi ^{2}/6\lambda ^{2}$, where $\lambda$ is a scale parameter. Given these assumptions, the probability of the sequence of best-worst choices made by individual *n* can be represented by the multinomial logit (MNL) model:

2 $$\begin{aligned} \Pr \left( y_{n}|x_{n}\right) =\prod \limits _{t=1}^{T_{n}}\dfrac{\exp \left\{ \lambda \left[ \left( \beta x_{b_{nit}} + \gamma _{b_{nit}}\right) - \left( \beta x_{w_{nit}} + \gamma _{w_{nit}}\right) \right] \right\} }{\sum \nolimits _{j=1}^{J}\exp \left\{ \lambda \left[ \left( \beta x_{b_{njt}} + \gamma _{b_{njt}}\right) -\left( \beta x_{w_{njt}} + \gamma _{w_{njt}}\right) \right] \right\} }, \end{aligned}$$

where $y_{n}$ gives the sequence of best-worst choices over the $T_{n}$ BWS tasks for respondent *n*, i.e., $y_{n}=\left\langle i_{n1},i_{n2},\ldots ,i_{nT_{n}}\right\rangle$. However, the scale factor, $\lambda$ is typically unidentifiable due to confounding with the vector of parameters. For this reason, it is usually arbitrary set it to one, leading to a constant variance equal to $\pi ^{2}/6$.

The MNL model is based on the very strong assumption that all respondents hold the same preferences. For this reason, we move to a random parameters logit (RPL) model that can accommodate heterogeneity in respondents’ preferences. In this specification, the vector $\tilde{\beta }_{n}$ is treated as continuously distributed random terms entering the utility function. However, it is clearly not possible to know $\beta _{n}$ with certainty for each respondent *n*. For this reason, in estimation, we accommodate heterogeneity across respondents by allowing for random variation. Denoting the joint density of $\left[ \beta _{n1},\beta _{n2},\ldots ,\beta _{nK}\right]$ by $f\left( \Theta _{n}|\Omega \right)$, where $\Theta _{n}$ represents the vector comprised of the random parameters, and $\Omega$ denotes the parameters of these distributions (e.g., the mean and variance), the unconditional choice probability is the integral of the MNL formula over all possible values of $\tilde{\beta }_n$:

3 $$\begin{aligned} \Pr \left( y_{n}|x_{n},\Omega \right) =\int \prod \limits _{t=1}^{T_{n}}\dfrac{\exp \left\{ \lambda \left[ \left( \tilde{\beta }_{n} x_{b_{nit}} + \tilde{\gamma }_{b_{nit}}\right) - \left( \tilde{\beta }_{n} x_{w_{nit}} + \tilde{\gamma }_{w_{nit}}\right) \right] \right\} }{\sum \nolimits _{j=1}^{J}\exp \left\{ \lambda \left[ \left( \tilde{\beta }_{n} x_{b_{njt}} + \tilde{\gamma }_{b_{njt}}\right) -\left( \tilde{\beta }_{n} x_{w_{njt}} + \tilde{\gamma }_{w_{njt}}\right) \right] \right\} }f\left( \Theta _{n}|\Omega \right) \mathrm {d}\left( \Theta _{n}\right) . \end{aligned}$$

The choice probabilities in this RPL model cannot be calculated exactly (because the integrals do not have a closed form). Instead, they have to be approximated through simulating the log-likelihood with *R* quasi-random draws.

#### Trio-wise modeling approach

**Fig. 5:**
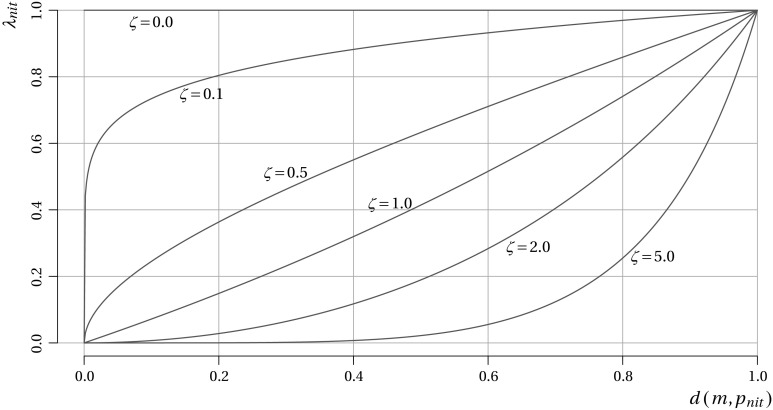
Role of $\zeta$ on $\lambda _{nit}$

Our proposed trio-wise method follows a similar modeling approach. However, the fundamental difference is that we are now in a position to explicitly recognize the strength of preferences within the model. Recall that preference intensity is reflected by the distance between the chosen point and the centroid, $d ( m,p )$. The selected points that are close to the centroid imply that the probabilities of the three items being most/least preferred are quite similar. As distance from the centroid increases, the differences in the probabilities are expected to become more profound. Remark that this bears resemblance to the role of the scale factor, $\lambda$ (i.e., as $\lambda$ increases the probabilities become more divergent). Therefore, in the trio-wise case, $\lambda$ can be expressed as a function of this distance, which we present as the following:

4 $$\begin{aligned} \lambda _{nit} = \exp \left[ \ln (c+1)d\left( m,p_{nit}\right) ^\zeta \right] -1, \end{aligned}$$

where $\lambda _{nit}$ is the scale parameter that takes a value between 0 and the specified constant *c* (where $c>0$), defined as a function of the normalized Euclidean distance, $0\le d ( m,p_{nit} ) \le 1$ (i.e., where the altitude of the triangle is set to 1.5), and where $\zeta$ is an estimated (non-negative) coefficient. For the purpose of estimation, the constant *c* can be set to one, so that $0\le \lambda _{nit} \le 1$. When $d ( m,p_{nit} ) =0$ (i.e., when the centroid is the chosen point), $\lambda _{nit}=0$, meaning that the probabilities associated with each item are the same. However, when $d ( m,p_{nit} ) = 1$ (i.e., when a vertex is clicked), $\lambda _{nit}=1$, which equates to a higher predicted choice probability for the associated item. Note that the values of $\lambda _{nit}$ in the interval $0< d ( m,p_{nit} ) < 1$ are determined by the estimated value of $\zeta$, as illustrated in Fig. 5. Thus, $\zeta$ defines the extent to which preference intensity is accounted for in the model. If $\zeta$ is found to be zero, the model reverts back to the situation where $\lambda _{nit}=1\forall d ( m,p_{nit})$, which is analogous to the BWS specification. However, if $\zeta >0$, more similar probabilities are retrieved for items when there are relatively weak preference intensities (i.e., points chosen close to the centroid) compared to those recovered when strong preference intensities are exhibited (i.e., when the location clicked is relatively distant from the centroid). As $\zeta$ increases, the smaller the value of $\lambda _{nit}$ at the respective distance. This means that no restrictions are imposed on the linearity/non-linearity of the impact of distance on the scale parameter. The relationship can be concave, convex, or approximately linear. This is what makes the functional form of Eq. 4 ideally suited for measuring the relationship between the scale parameter and the Euclidean between the chosen point and the centroid.

In addition to the unique ordinal ranking of items (i.e., the six triangles formed by partitioning via the medians), two- and three-way ties need to be accommodated in the model. As illustrated in Fig. 1, there are six two-way ties. Three of these relate to the case where one item is deemed superior over two equally ranked items. Such a ranking is expressed when a point is chosen along one of the three segments joining the centroid to the vertices, which would denote either $A>B=C$, $B>A=C$ or $C>A=B$. The utility functions for these alternatives can be written as:

5a $$\begin{aligned} U_{nit}= \underbrace{\left( \beta x_{b_{nit}} + \gamma _{b_{nit}}\right) }_{\text {Best}}-\underbrace{\left[ \left( \beta x_{w_{nit}^{1}} + \gamma _{w_{nit}^{1}}\right) +\left( \beta x_{w_{nit}^{2}} + \gamma _{w_{nit}^{2}}\right) \right] /2}_{\text {Worst}}+\varepsilon _{nit}, \end{aligned}$$

where ${w_{nit}^{1}}$ and ${w_{nit}^{2}}$ associate the two items in the choice task that are equally least preferred. The three remaining two-way ties describe the situation where a respondent considers two of the items as being equally preferred compared to an item that they regard as being inferior. This occurs when a point is selected along one of the three segments joining the centroid and the midpoints opposite the vertices, leading to either $A=B>C$, $A=C>B$ or $B=C>A$ and the following utility expression:

5b $$\begin{aligned} U_{nit}= \underbrace{\left[ \left( \beta x_{b_{nit}^{1}} + \gamma _{b_{nit}^{1}}\right) +\left( \beta x_{b_{nit}^{2}} + \gamma _{b_{nit}^{2}}\right) \right] /2}_{\text {Best}}-\underbrace{\left( \beta x_{w_{nit}} + \gamma _{w_{nit}}\right) }_{\text {Worst}}+\varepsilon _{nit}, \end{aligned}$$

where ${b_{nit}^{1}}$ and ${b_{nit}^{2}}$ associate the two items in the choice task that are equally most preferred. There is also a single three-way tie in the three items, which is revealed when the centroid is clicked. In this case, the average of the representative utility becomes zero, which yields equal choice probabilities for the alternative options.

#### Ratio-scaled probabilities

The vector of estimated utility coefficients, $\beta$, in the above models are on an interval scale and typically consist of both negative and positive values, making their interpretation difficult. For this reason, similar to Campbell and Erdem [13], it is useful to convert the raw coefficients, which are zero-centered, to ratio-scaled probabilities, which we denote using $\mathop {\mathrm{{Pr}}}\limits ^{*}\left( x\right)$. For item *k*, the conversion to a 0–100 point ratio scale is achieved as follows:

6a $$\begin{aligned} \mathop {\mathrm{{Pr}}}\limits ^{*}\left( x_{k}\right) =\left( \dfrac{\exp \left( \lambda \beta _{k}\right) }{\exp \left( \lambda \beta _{k}\right) +S-1}/ \sum \limits _{k=1}^{K} \dfrac{\exp \left( \lambda \beta _{k}\right) }{\exp \left( \lambda \beta _{k}\right) +S-1} \right) \times 100, \end{aligned}$$

where *S*, as previously defined, is the number of items shown per choice task. These ratio-scaled probabilities provide an intuitive interpretation since we can say that an item with a score of 20 is twice as preferred or important as an item with a score of 10. Note that individual-specific ratio-scaled probabilities can also be retrieved to allow differences in preferences due to individual characteristics to be assessed. For this, we use Bayes’ theorem:

6b $$\begin{aligned} \mathbb {E}\left( \mathop {\mathrm{{Pr}}}\limits ^{*}\left( x_{k_{n}}\right) \right) = \sum \limits _{r=1}^{R}\dfrac{\Pr \left( y_{n}|x_{n},\beta _{r},\gamma _{b},\gamma _{w},\zeta \right) \mathop {\mathrm{{Pr}}}\limits ^{*}\left( x_{k}|\beta _{r},\bar{\lambda }_{nit}\right) }{\sum \nolimits _{r=1}^{R}\Pr \left( y_{n}|x_{n},\beta _{r},\gamma _{b},\gamma _{w},\zeta \right) }, \end{aligned}$$

where $\mathbb {E}(\mathop {\mathrm{{Pr}}}\limits ^{*}(x_{k_{n}}))$ represents the expected value of the ratio-scaled probability for respondent *n* for item *k*, and where $\Pr \left( y_{n}|x_{n},\beta _{r},\gamma _{b},\gamma _{w},\zeta \right)$ denotes the probability of observing the sequence of choices by respondent *n* given $x_{n}$ and the values of $\beta _{r}$, $\gamma _{b}$, $\gamma _{w}$ and $\zeta$. Here $\beta _{r}$, with $r=1,\ldots ,R$, represents an independent random draw with equal weight from $f({\Theta }|\hat{\Omega })$, and $\mathop {\mathrm{{Pr}}}\limits ^{*}\left( x_{k}|\beta _{r}\right)$ gives the ratio scaled-probability for item *k* given the values of $\beta _{r}$ and the value of $\bar{\lambda }_{nit}$ (derived using the mean distance of respondent *n*’s choices from the centroid). Note that these conditional parameters themselves follow a distribution, Eq. 6b merely gives the expected value of these distributions (e.g., see Hess [36] for further details). Nevertheless, this does give us some information about the most likely position of a respondent on the distributions of ratio-scaled probabilities, which is of greatest interest.

## Results

We begin this section with a rudimentary examination of the choices and response latency in both survey treatments. Following this, we report estimation results from our MNL and RPL models and post-estimation analysis.

### Examination of choices and response time

An examination of all 15,129 best-worst choice observations reveals that the characteristics located at the top of the BWS choice task have a higher likelihood of being chosen as the ‘best’ characteristic (34%), as compared to the items located at the bottom of the choice task (31%). The reverse is observed for the worst choices. Respectively, other things being equal, the top and bottom characteristics listed in the BWS choice task were identified as being the least preferred in 31 and 35% of cases. Similar BWS position effects were found in Campbell and Erdem [13].

**Fig. 6:**
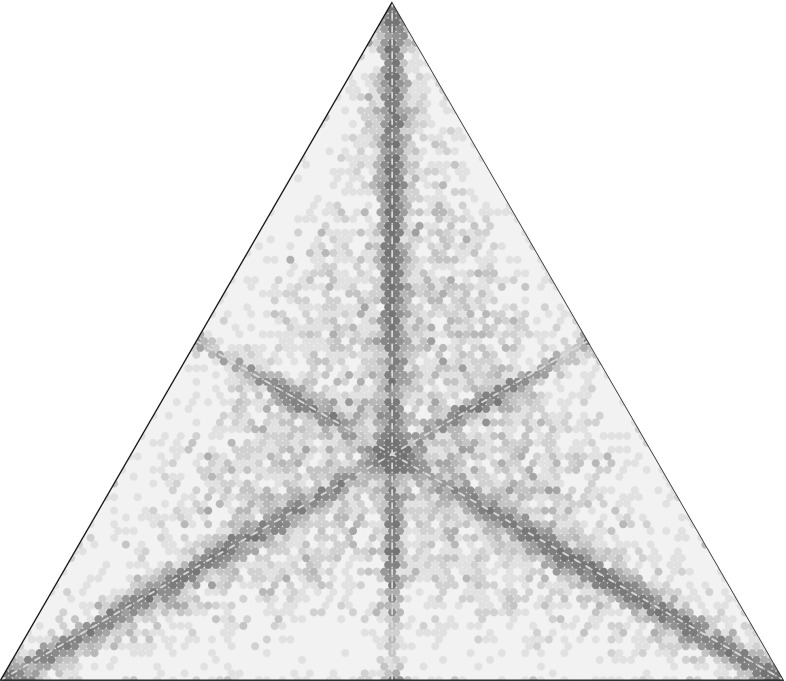
Where people clicked in the trio-wise survey

An examination of the trio-wise observations is performed using the smoothed density representation of the locations of all 15,381 choice observations, as shown in Fig. 6. First of all, we see a good spread in the locations clicked. This is an important finding. It gives a clear signal that there is heterogeneity in preference intensities across respondents. This heterogeneity also highlights a weakness of the BWS approach, which is its inability of capturing any measure of preference intensity.

Another interesting finding is that a comparison of the density of choice locations in the six triangles formed by partitioning by the medians suggests that the trio-wise choice may also be subject to a position bias. In particular, all else being held constant, there is a seemingly increased tendency for respondents to click closer to the top vertex, followed by the bottom-right vertex. This issue aside, it also apparent that in many cases respondents hold relatively weak preferences for the different characteristics (ca. 65% of choices are within the inscribed circle). This is not a surprising result, given that respondents were answering stated preference questions for a complex problem, and is likely to be something which many of them will have given little thought to before completing the survey (i.e., only 2% stated they had engaged in such activities).

As apparent from the darker shades along (or near to) the angle bisectors, a further intriguing result is the number of choices representing ties. In fact, these accounted for just over one-quarter of the choices. We find that 26% of choices were two-way ties and less than 1% were three-way ties. This gives a clear signal of preference indifference between the characteristics shown in the choice tasks. Such a pattern indicates that forcing respondents to provide a complete ordinal ranking, as is the case in BWS, may not be appropriate (especially in situations where respondents may hold relatively weak preferences and perhaps had little prior knowledge). Inspecting the trio-wise choices further, we find that less than 1% of respondents always selected a point along an angle bisector and almost 60% of respondents only selected a point along an angle bisector in two or fewer choice tasks. This reassures us that respondents were not necessarily choosing along the angle bisectors as a simplifying heuristic.

In an attempt to gauge the cognitive effort invested by respondents, we use the length of time respondents required to answer the choice tasks as a proxy for choice difficulty (such that longer response latencies would indicate greater difficulty than shorter response latencies). In “Examination of choices and response time” we present back-to-back histograms depicting the length of time per choice task for each treatment. Across both stated preference elicitation methods, the response latency results are remarkably consistent, meaning that we can be relatively assured that the cognitive burden placed on respondents who completed the trio-wise choice tasks was no greater to that placed on those who answered the BWS treatment. In line with evidence presented elsewhere (e.g., [37, 38]), respondents spent considerably longer completing the first few choice tasks, which signifies learning.

Response latency for best-worst scaling and trio-wise methods by choice task

### Estimation results

The public’s allocation of importance to various characteristics of involvement activities in BWS and trio-wise surveys are investigated under the MNL and the RPL specifications using the OxMetrics software [39]. In the RPL models, the choice probabilities are approximated by simulating the log-likelihood with 500 quasi-random Sobol draws. Specification of random parameters requires the assumption of adequate distribution functions for each of the random parameters in the utility [40, 41]. After evaluating the results from various specifications and distributional assumptions, we specify all parameters within the vector $\beta$ as having Normal distributions: $\beta _{k} = \mu _{k} + \sigma _{k}\upsilon _{k}$, where $\upsilon _{k}$ is an independent standard Normal deviate and $\mu _{k}$ and $\sigma _{k}$ are parameters to be estimated, which can be interpreted as the mean and the standard deviation respectively of the *k*th Normally distributed parameter.

#### Best-worst scaling results

We start our analysis with the baseline MNL model that assumes homogeneous preferences. According to the results presented in Table 1, respondents regard impact, followed by focus, to be the most important features of public involvement activities. The format of public involvement activities along with who delivers them and where, how often and how long they happen are all deemed to be relatively less important aspects.

**Table 1:** Estimation results of the best-worst scaling data

	MNL	RPL
$\hat{\mu }_{\text {format}}$	−0.325***0.016)	−0.582*** (0.031)
$\hat{\mu }_{\text {who}}$	−0.339*** (0.016)	−0.590*** (0.039)
$\hat{\mu }_{\text {where}}$	−0.523*** (0.016)	−0.930*** (0.038)
$\hat{\mu }_{\text {howoften}}$	−0.228*** (0.016)	−0.413*** (0.030)
$\hat{\mu }_{\text {howlong}}$	−0.511*** (0.016)	−0.850*** (0.033)
$\hat{\mu }_{\text {impact}}$	1.297*** (0.022)	2.337*** (0.055)
$\hat{\mu }_{\text {focus}}$	0.682*** (0.017)	1.081*** (0.034)
$\hat{\sigma }_{\text {format}}$		0.904***(0.035)
$\hat{\sigma }_{\text {who}}$		1.334*** (0.043)
$\hat{\sigma }_{\text {where}}$		1.220*** (0.040)
$\hat{\sigma }_{\text {howoften}}$		0.850*** (0.038)
$\hat{\sigma }_{\text {howlong}}$		0.992*** (0.039)
$\hat{\sigma }_{\text {impact}}$		1.479*** (0.051)
$\hat{\sigma }_{\text {focus}}$		0.932*** (0.039)
$\hat{\gamma }_{b_{A}}$	0.043*** (0.014)	0.083*** (0.018)
$\hat{\gamma }_{b_{B}}$	0.021* (0.015)	0.046*** (0.019)
$\hat{\gamma }_{w_{A}}$	0.010 (0.014)	0.026* (0.017)
$\hat{\gamma }_{w_{B}}$	−0.007 (0.014)	−0.014 (0.018)
Log-likelihood	−22,901.133	−20,620.708
*K*	11	18
$\bar{\rho }^{2}$	0.155	0.239
AIC	45,824.267	41,277.416
BIC	45,908.135	41,414.655

Standard errors in parentheses. *p < 0.10; **p < 0.05; ***p < 0.001. For identification purposes, $\beta _{\text {cost}}$, $\hat{\gamma }_{b_{C}}$ and $\hat{\gamma }_{w_{C}}$ are arbitrarily set as the base levels

The position-specific constants retrieved under the MNL model give an important insight into potential position effects. We draw attention to the fact that these constants are non-zero, and the constant representing the top position for the best choice (denoted by $\hat{\gamma }_{b_{A}}$) is positive and significantly different with respect to the baseline (which is the bottom position). This means that, all else held constant, respondents are significantly more likely to identify the characteristic of public involvement presented at the top of a BWS task as being the most important characteristic (irrespective of what the characteristic is). Although not significant, the fact that position-specific constants associated with the worst choices are not the same signifies that the schematic cues stemming from characteristic’s position may not be the same for most and least important choices. This is consistent with findings in Campbell and Erdem [13].

Moving to the RPL model, the implicit ranking of the features of public involvement implied by the estimated means of the random parameters are the same as those deduced from the MNL model. However, the RPL model reveals a high degree of preference heterogeneity, as evident from the estimated standard deviations. All standard deviations are found to be highly significant, and in almost all cases are higher than their respective means (in absolute terms) and can, therefore, be considered as high-variance. Not surprisingly, the RPL model yields a much better model fit—indeed, there is an improvement by almost 300 log-likelihood units at the expense of just seven additional estimated parameters. A comparison of the $\bar{\rho }^{2}$ and information criteria statistics confirm this finding even after accounting for the loss of parsimony. Looking at the position-specific constants attained under the RPL model, we, again, observe some position effects. Interestingly, we now find evidence that, all else being equal, a feature of public involvement located in either the top or middle position is significantly more likely to be selected as being most important compared to the feature located at the bottom of a BWS tasks. We note that no significant position-effects are observed for the worst choices.

#### Trio-wise results

The BWS results provide a benchmark against which we test the trio-wise results for convergent validity. Therefore, for the trio-wise data we consider the same MNL and RPL specifications, but where the two- and three-way ties are accommodated. For comparison, we also present results where the scale parameter is fixed to 1 (i.e., where $\zeta$ = 0 in Eq. 4) and the scale parameter is not fixed but expressed as a function of the distance between the centroid and the chosen point (i.e., where $\zeta \ne 0$ in Eq. 4). The results of these models are presented in Table 2.

**Table 2:** Estimation results of the trio-wise data

	MNL		RPL	
	$\hat{\zeta }=0$	$\hat{\zeta }\ne 0$	$\hat{\zeta }=0$	$\hat{\zeta }\ne 0$
$\hat{\mu }_{\text {format}}$	−0.208*** (0.016)	−0.345*** (0.025)	−0.288*** (0.022)	−0.571*** (0.041)
$\hat{\mu }_{\text {who}}$	−0.249*** (0.016)	−0.371*** (0.025)	−0.362*** (0.028)	−0.680*** (0.053)
$\hat{\mu }_{\text {where}}$	−0.107*** (0.016)	−0.161*** (0.024)	−0.157*** (0.026)	−0.331*** (0.050)
$\hat{\mu }_{\text {howoften}}$	−0.171*** (0.017)	−0.292*** (0.026)	−0.240*** (0.024)	−0.510*** (0.044)
$\hat{\mu }_{\text {howlong}}$	−0.239*** (0.016)	−0.372*** (0.025)	−0.328*** (0.024)	−0.608*** (0.044)
$\hat{\mu }_{\text {impact}}$	0.646*** (0.017)	1.008*** (0.033)	0.880*** (0.031)	1.690*** (0.070)
$\hat{\mu }_{\text {focus}}$	0.287*** (0.016)	0.447*** (0.025)	0.364*** (0.027)	0.667*** (0.052)
$\hat{\sigma }_{\text {format}}$			0.532*** (0.030)	0.912*** (0.055)
$\hat{\sigma }_{\text {who}}$			0.805*** (0.032)	1.472*** (0.066)
$\hat{\sigma }_{\text {where}}$			0.748*** (0.031)	1.465*** (0.067)
$\hat{\sigma }_{\text {howoften}}$			0.558*** (0.032)	1.001*** (0.061)
$\hat{\sigma }_{\text {howlong}}$			0.575*** (0.031)	1.071*** (0.059)
$\hat{\sigma }_{\text {impact}}$			0.923*** (0.034)	1.781*** (0.074)
$\hat{\sigma }_{\text {focus}}$			0.776*** (0.031)	1.482*** (0.066)
$\hat{\gamma }_{b_{A}}$	0.232*** (0.017)	0.291*** (0.022)	0.263*** (0.018)	0.433*** (0.031)
$\hat{\gamma }_{b_{B}}$	−0.060*** (0.017)	0.354*** (0.026)	−0.067*** (0.018)	−0.112*** (0.030)
$\hat{\gamma }_{w_{A}}$	−0.131*** (0.017)	−0.105*** (0.024)	−0.128*** (0.018)	−0.297*** (0.031)
$\hat{\gamma }_{w_{B}}$	0.144*** (0.017)	−0.247*** (0.027)	0.161*** (0.018)	0.339*** (0.031)
$\hat{\zeta }$		0.264*** (0.026)		0.388*** (0.017)
Log-likelihood	−38,130.816	−37,946.767	−36,784.190	−36,345.381
*K*	11	12	18	19
$\bar{\rho }^{2}$	0.033	0.038	0.067	0.078
AIC	76,283.631	75,917.533	73,604.380	72,728.762
BIC	76,367.681	76,009.224	73,741.916	72,873.939

Standard errors in parentheses. *p < 0.10; **p < 0.05; ***p < 0.001. For identification purposes, $\beta _{\text {cost}}$, $\hat{\gamma }_{b_{C}}$ and $\hat{\gamma }_{w_{C}}$ are arbitrarily set as the base levels

Beginning with the results obtained under the MNL models we find many similarities to those already reached from the BWS data. In particular, the same general implicit importance ranking of public involvement activities is observed: respondents, again, reveal that the format of the activity, who delivers it, where, how often and how long it happens are of lesser importance compared to the impact, focus and cost. This is an important finding, since it confirms that both data sources yield consistent information on the underlying preferences for public involvement activities. We also remark the large improvement in model fit achieved by specifying the scale parameter as a function of the distance between the centroid and the chosen point. The estimated value of $\zeta$, which defines the role of preference intensity in the model, is statistically significant. This means that we can reject the null hypothesis that preference intensity has no bearing on choice probabilities. This is a further crucial finding since it indicates that our trio-wise approach passes this important internal validity test (i.e., choice tasks where respondents exhibited strong preferences are associated with lower error variance compared to those where their strength of preferences is weaker). Comparing the estimated value of $\zeta$ against those illustrated in Fig. 5 reveals that the similarities in predicted choice probabilities diminish quite abruptly with distance from the centroid.

The results from two MNL models also show significant position-specific constants. This is not surprising given the observed choices, as presented in Fig. 6. Specifically, it is found that respondents are most likely to select a point closer to the top vertex (areas 1 and 6 in Fig. 1) irrespective of the characteristic of public involvement activity, and least likely to select a point located closer to the bottom left vertex (areas 4 and 5 in Fig. 1).

As we turn our attention to the RPL models we, once more, find compelling evidence of preference heterogeneity. Regardless of whether or not preference intensities are directly accommodated in the model, all the standard deviations are significant. In addition, similar to what we observed from the BWS data, these are found to be quite large relative to their respective means. This serves as another convergent validity check, and further corroborates our trio-wise approach for stated preference elicitation. Again, allowing for this preference heterogeneity leads to huge improvements in model fit. Of greater interest, however, is the large jump in model fit (by over 400 log-likelihood units) achieved when the scale parameter is specified to be dependent on the distance between the centroid and the chosen point. In this case, the estimated value of $\zeta$ is slightly higher compared to that derived under the MNL counterpart. This signifies that the resemblance in predicted choice probabilities at chosen points closer to the centroid may not be as pronounced as implied under the MNL model. The position-specific constants obtained under the RPL models provide the same inferences to those reached under the MNL models.

#### Post estimation results

**Fig. 7:**
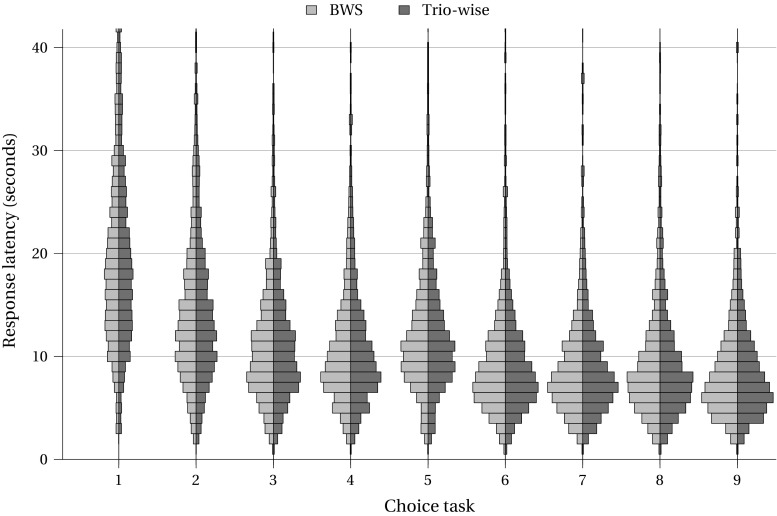
Response latency for best-worst scaling and trio-wise methods by choice task

To ease interpretation and allow comparisons to be made between preferences elicited from the BWS approach and the trio-wise approach, in Fig. 7 we present boxplots of the means of the conditional (individual-specific) ratio-scaled probabilities retrieved from the RPL models (as described in Eq. 6). The boxplots show the median, the 25th and 75th percentile points shown by ‘hinges’, outliers and the means of the distributions presented with white circles. Notches are drawn to show the 95% confidence interval of the median.

As can be seen, both stated preference elicitation techniques produce similar distributions of ratio-scaled probabilities. This said, there are a few noticeable differences. The most salient difference is that the differences in scores produced from the BWS data are more apparent. Compare, for instance, the median scores estimated for the activity impact and where the activity takes place. The BWS data suggests that impact is perceived to be, on average, almost five times more important compared to where it happens. However, in both trio-wise models, impact is found to be less than twice as important. We acknowledge that the conversion to ratio-scaled probabilities does not factor out the scaling of the parameter estimates that is related to the scale factor of the unobserved Gumbel error component, which we admit confines any meaningful comparison of the ratio-scaled probabilities between models and datasets. Notwithstanding this limitation, we do feel that the starker differences in ratio-scaled probabilities are, to some extent at least, an artefact of the forced choice nature of the BWS choice tasks.

The trio-wise approach accommodates situations where respondents are indifferent between two or more of the items presented in the choice task. Not surprisingly, this leads to less extreme scores. Note also that respondents express their preference intensities in the trio-wise choice tasks, which will also partially explain why the differences in the characteristics are less pronounced. Indeed, while both stated preference elicitation techniques provide convincing evidence for prioritizing the impact of public involvement activities over all other aspects of public involvement, it may not be to the extent suggested by the BWS data.

**Fig. 8:**
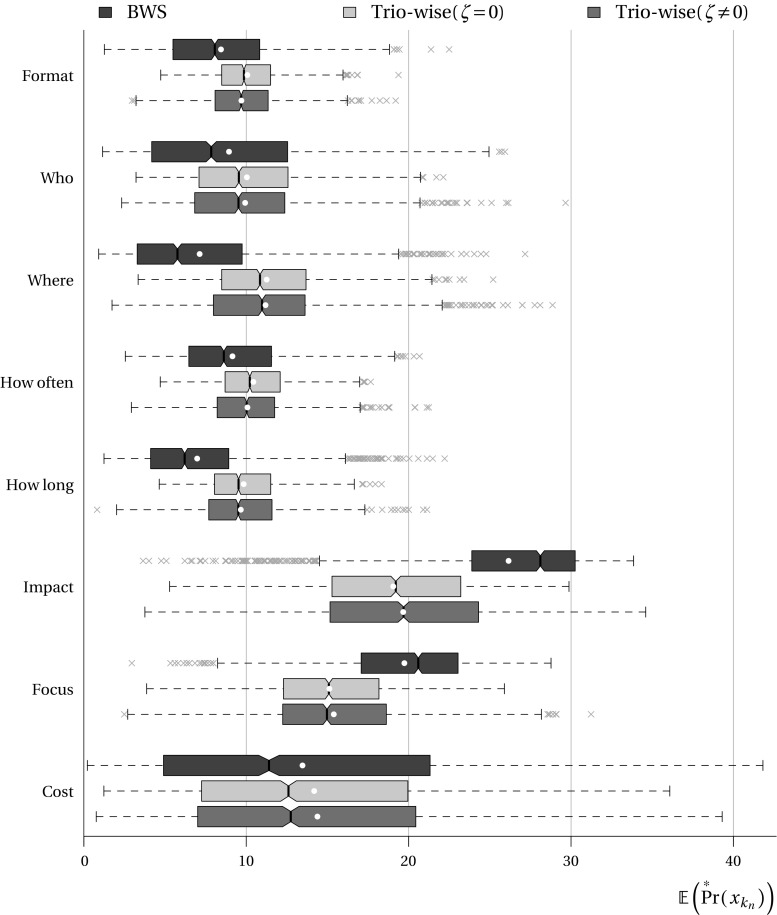
Comparison of boxplots of the means of the conditional ratio-scaled probabilities derived from the best-worst scaling and trio-wise random parameters logit models

As part of our post estimation analysis we derived summary statistics of the individual-specific ratio-scaled probabilities according to a number of socio-economic characteristics (e.g., age, gender, employment, education categories). No significant differences were observed, so we, therefore, do not present the results. This was consistent in both BWS and trio-wise cases, which is an additional indication of similarity between both stated preference techniques (Fig. 8).

## Conclusions

Various stated preference elicitation methods can be used to rank the characteristics of public policy in terms of their importance to the public. One such technique, which is widely used to prioritize resources in policies relating to health, transport and the environment, is the best-worst scaling (BWS) approach.

In this paper, we propose an alternative stated preference elicitation, which we term ‘trio-wise’. This new technique shares many of the same characteristics of BWS, but, importantly, allows respondents to express a measure of preference intensity, which is not captured in BWS. The key feature of this approach is that the choice task is represented as an equilateral triangle and respondents are allowed to click on any point on the triangle that best represents their rankings of the items presented to them. Crucially, the additional insight relating to the strength of respondents’ preferences means that the same level of information can be recouped from respondents using fewer choice tasks. Moreover, the way trio-wise designed also allows for preference indifferences by permitting respondents to choose points that are equidistant to all or two of the vertex.

At the heart of this paper is a comparison of the BWS approach and our trio-wise technique, which are used to uncover the preferences among the UK general public for ways to involve them in decisions affecting the national health care system. The two stated preference elicitation methods are compared in terms of preference rankings and policy repercussions. Using multinomial logit and random parameters logit modeling frameworks, we find that our trio-wise approach produced results that are remarkably similar to those obtained from the traditional BWS. This is an important finding, since it increases our confidence of the convergent validity of the trio-wise approach, in that it is capable of retrieving consistent information on the underlying preferences for public involvement activities. The fundamental difference is that the preference intensity can be explicitly accounted for when modeling the trio-wise choices. We show that this additional insight is advantageous, as evident from the large improvements in model fits achieved when the scale parameter is represented by a function of preference intensity. In particular, we find significantly lower error variance for choice tasks where respondents exhibited strong preferences compared to choice tasks where their strength of preferences was evidently weaker. This result demonstrates internal validity and, therefore, further underpins the trio-wise approach as a reliable method for preference elicitation. Notwithstanding the similarities, the differences in ranks for the characteristics of public involvement activities retrieved from the BWS data are somewhat more pronounced. We attribute these differences to the fact that the BWS technique does not capture either any measure of the strength of preferences nor situations where respondents are indifferent between two or more of the characteristics presented in the choice task.

There are also clear policy implications of the preference rankings reported in this study. Understanding how the public prioritizes the features of involvement activities will not only help increase engagement with different groups in society but should result in better-informed policies that meet the public’s expectations. Hitherto, such an elicitation of the importance of different ways to involve the public in health care decisions has been missing. This case study, therefore, makes an important contribution in this area. Thus, the results can be used to inform decisions on how to best involve the public in health care decisions. Based on our results, we find significant heterogeneity in how the UK public prioritize involvement activities in health care. Interestingly, however, we do not find any evidence of different priorities among different socio-economic groups. On average, the impact of the activity is found to be of greatest importance. The general public also consider the focus and cost of the activity to be highly important. Other features of public involvement activities—including their impact, who leads them, where they happen, how often they happen and how much time they require—are found to be of lesser importance to the public.

The trio-wise approach signals clear evidence of heterogeneity in preference intensities, none of which is explained by the BWS data. Similarly giving respondents the opportunity to exhibit indifferent preferences is important as it may avoid respondents feeling forced into providing an ordinal ranking when their preferences are indistinguishable. Both these aspects are likely to be especially important when respondents are asked to state their preferences relating to aspects of public health. Prioritizing features of public health is complex and involves many uncertainties. Moreover, few respondents will have given any thought to ranking these aspects before completing the survey. Indeed, given that the formation of rational, consistent and well-formed preferences are formed due to experience [42], in such cases it is perhaps unreasonable to expect respondents to express strong preferences and to be able to differentiate between all these interconnected aspects. As our findings suggest, the concern is that not giving respondents the opportunity to express either their preference intensity or indifference could lead to misleading results. Importantly, the trio-wise method does not appear to be any more burdensome to respondents. In fact, there is even some anecdotal evidence suggesting that respondents prefer the trio-wise elicitation questions over the BWS questions. Feedback gathered from respondents after they completed the trio-wise questionnaire were positive, with statements such as “enjoyable”, “interesting”, and “useful and easy way to convey how I felt rather than tick boxes”. We acknowledge, however, that further testing, including the use of face-to-face interviews, in different contexts would be helpful to corroborate this.

Despite our findings, we recognize the need for further research. First and foremost, an investigation into the suitability (and restrictions) of the trio-wise assumptions is warranted. This is needed to provide a clearer insight into the consequences (and potential biases) of these assumptions for preference elicitation. Specifically, the implications of the necessity of three alternatives should be investigated. The potentially unfamiliar nature of the trio-wise choice tasks and the degree to which this affects respondent’s ability to provide a demand revealing choice also needs to be explored. Furthermore, research is needed to assess whether our findings apply in other settings as well as how our trio-wise approach stacks up against other preference elicitation approaches where the number of alternatives are not restrained. By comparing against the BWS method, we were able to use the exact same experimental design in the trio-wise method (in terms of number of alternatives per choice task and number of choice tasks). This would not have been possible had we chosen visual analogue scale or pair-wise methods as a comparator, since they all use a form of a linear scale between two end-points. Therefore, we would not have been able to distinguish between the design and method effects. Moreover, some of these other methods do not as readily lend themselves to the same random utility theoretical framework. Indeed, we feel that if a different comparator had been used, comparisons would have been difficult (if not impossible) to make since we would not be able to say the extent to which they are due the approach or the modeling framework. Nevertheless, we admit that a comparison with other methods would give a more definitive insight into the relative merits of the trio-wise method, which warrants further research. Relatedly, a comparison against more widely used and established stated preference elicitation methods would help provide further external validation. Extending the trio-wise method to include attributes and levels (analogous to the multi-profile BWS Case 3) offers an interesting avenue for future research, even though it is likely to lead to a more challenging choice. The suitability of the approach for welfare estimation (e.g., marginal willingness to pay) also needs careful assessment. We acknowledge that further research on respondents’ cognition and their understanding of the trio-wise choice task and any heuristics they adapt when reaching their choices is also needed.

## Footnotes

1 Strictly speaking, the trio-wise choice task consisted of 5151 possible locations that the respondent could click, which is sufficiently large to allow respondents to precisely make their choice.

2 We recognize that this makes it difficult to corroborate our claim that fewer choice tasks are needed under the trio-wise approach to gather the same level of information.

## References

[1] Finn A, Louviere J (1992). Determining the appropriate response to evidence of public concern: the case of food safety. J. Public Policy Mark..

[2] Thurstone LL (1927). A law of comparative judgment. Psychol. Rev..

[3] Cohen S, Orme B (2004). Asking survey respondents about their preferences creates new scaling decisions. Mark. Res..

[4] Rasmussen, J.L.: Situational judgment test responding: best and worst or rate each response. Ph.D. thesis, Texas A&M University (2009)

[5] Flynn TN, Louviere JJ, Peters TJ, Coast J (2007). Best-worst scaling: what it can do for health care research and how to do it. J. Health Econ..

[6] Potoglou D, Burge P, Flynn T, Netten A, Malley J, Forder J, Brazier JE (2011). Best-worst scaling vs. discrete choice experiments: an empirical comparison using social care data. Soc. Sci. Med..

[7] Marti J (2012). A best-worst scaling survey of adolescents’ level of concern for health and non-health consequences of smoking. Soc. Sci. Med..

[8] Lancsar E, Louviere J, Donaldson C, Currie G, Burgess L (2013). Best worst discrete choice experiments in health: methods and an application. Soc. Sci. Med..

[9] Erdem S, Rigby D (2013). Investigating heterogeneity in the characterization of risks using best worst scaling. Risk Anal..

[10] Torrance GW, Feeny D, Furlong W (2001). Visual analog scales do they have a role in the measurement of preferences for health states?. Med. Decis. Mak..

[11] Louviere JJ, Flynn TN (2010). Using best-worst scaling choice experiments to measure public perceptions and preferences for healthcare reform in Australia. Patient Patient Centered Outcomes Res..

[12] Scarpa R, Notaro S, Louviere J, Raffaelli R (2011). Exploring scale effects of best/worst rank ordered choice data to estimate benefits of tourism in Alpine grazing commons. Am. J. Agric. Econ..

[13] Campbell D, Erdem S (2015). Position bias in best-worst scaling surveys: a case study on trust in institutions. Am. J. Agric. Econ..

[14] Louviere JJ, Flynn TN, Marley A (2015). Best-worst Scaling: Theory, Methods and Applications.

[15] Campbell D, Boeri M,  Dohery E, Hutchinson WG (2015). Doherty, Hutchinson, W. G.: Learning, fatigue and preference formation in discrete choice experiments. J. Econ. Behav. Organ..

[16] Kaplan RM, Feeny D, Revicki D (1993). Methods for assessing relative importance in preference based outcome measures. Qual. Life Res..

[17] Revicki DA, Kaplan RM (1993). Relationship between psychometric and utility-based approaches to the measurement of health-related quality of life. Qual. Life Res..

[18] Johannesson M, Jönsson B, Karlsson G (1996). Outcome measurement in economic evaluation. Health Econ..

[19] Health and Social Care Act 2012, Part 5 Public Involvement and Local Government, Chapter 1 Public Involvement. 2012. http://www.legislation.gov.uk/ukpga/2012/7/part/5/enacted. Accessed: 3 September 2015

[20] Health Canada: Public involvement framework. Health Products and Food Branch, Health Canada (2013)

[21] Erdem S, Thompson C (2014). Prioritising health service innovation investments using public preferences: a discrete choice experiment. BMC Health Serv. Res..

[22] Gillard S, Borschmann R, Turner K, Goodrich-Purnell N, Lovell K, Chambers M (2010). ‘What difference does it make?’ Finding evidence of the impact of mental health service user researchers on research into the experiences of detained psychiatric patients. Health Expect..

[23] Howe A, Delaney S, Romero J, Tinsley A, Vicary P (2010). Public involvement in health research: a case study of one NHS project over 5 years. Prim. Health Care Res. Dev..

[24] Milewa T, Harrison S, Ahmad W, Tovey P (2002). Citizens’ participation in primary healthcare planning: innovative citizenship practice in empirical perspective. Crit. Public Health.

[25] Morris MC, Nadkarni VM, Ward FR, Nelson RM (2004). Exception from informed consent for pediatric resuscitation research: community consultation for a trial of brain cooling after in-hospital cardiac arrest. Pediatrics.

[26] Wiseman V, Mooney G, Berry G, Tang KC (2003). Involving the general public in priority setting: experiences from Australia. Soc. Sci. Med..

[27] Barber R, Boote JD, Parry GD, Cooper CL, Yeeles P, Cook S (2012). Can the impact of public involvement on research be evaluated? A mixed methods study. Health Expect..

[28] Jarret, L.: The Patient Involvement Unit: A report on a study to evaluate patient/carer membership of the first NICE Guideline Development Groups. National Institute for Clinical Excellence (NICE) and the National Health Service (NHS), UK (2004)

[29] Ursu, I., Cowl, J.: Community membership of NICE groups producing public health guidance: report of an evaluation study. National Institute for Clinical Excellence (NICE) and the National Health Service (NHS), UK (2010)

[30] Crawford MJ, Rutter D, Manley C, Weaver T, Bhui K, Fulop N, Tyrer P (2002). Systematic review of involving patients in the planning and development of health care. BMJ.

[31] Boote, J.: Patient and public involvement in health and social care research: a bibliography. NIHR Research Design Service for Yorkshire and the Humber (2011)

[32] Mockford C, Staniszewska S, Griffiths F, Herron-Marx S (2012). The impact of patient and public involvement on UK NHS health care: a systematic review. Int. J. Qual. Health Care.

[33] Brett J, Staniszewska S, Mockford C, Herron-Marx S, Hughes J, Tysall C, Suleman R (2014). Mapping the impact of patient and public involvement on health and social care research: a systematic review. Health Expect..

[34] ChoiceMetrics: Ngene 1.1.1: User manual and reference guide. ChoiceMetrics, Sydney, Australia (2012)

[35] Manski CF (1977). The structure of random utility models. Theory Decis..

[36] Hess S (2010). Conditional parameter estimates from mixed logit models: distributional assumptions and a free software tool. J. Choice Model..

[37] Haaijer R, Kamakura W, Wedel M (2000). Response latencies in the analysis of conjoint choice experiments. J. Mark. Res..

[38] Rose JM, Black IR (2006). Means matter, but variance matter too: decomposing response latency influences on variance heterogeneity in stated preference experiments. Mark. Lett..

[39] Doornik JA (2007). Object-oriented matrix programming using Ox.

[40] Hensher DA, Greene WH (2003). The mixed logit model: the state of practice. Transportation.

[41] Hess S, Bierlaire M, Polak JW (2005). Estimation of value of travel-time savings using mixed logit models. Transp. Res. Part A Policy Pract..

[42] Plott CR, Arrow KJ, Colombatto E, Perlman M, Schmidt C (1996). Rational individual behaviour in markets and social choice processes: the discovered preference hypothesis. The Rational Foundations of Economic Behaviour.

